# Importance of Inclusive Education in General Medicine Through the Perception of Medical Trainees: A Thematic Analysis

**DOI:** 10.7759/cureus.47585

**Published:** 2023-10-24

**Authors:** Ryuichi Ohta, Takuji Katsube, Chiaki Sano

**Affiliations:** 1 Community Care, Unnan City Hospital, Unnan, JPN; 2 Family Medicine, Unnan City Hospital, Unnan, JPN; 3 Community Medicine Management, Faculty of Medicine, Shimane University, Izumo, JPN

**Keywords:** educator-learner relationship, community-based medical education, psychological safety, medical pedagogy, inclusivity, rural japan, thematic analysis, general medicine, family medicine

## Abstract

Background

General medical education plays a pivotal role in ensuring holistic care in the context of rapidly aging populations. Japan’s demographic trends underscore the significance of general medicine in elevating community care standards. Understanding and catering to the aspirations, perceptions, and ideals of medical students and residents can significantly augment the effectiveness of general medicine education. This research aimed to explore the perspectives of medical students on the ideal tenets of general medicine education in rural Japan.

Method

A qualitative approach was employed, focusing on medical students and residents with a keen interest in general medicine, all of whom underwent training at a rural-based Japanese hospital. Through semi-structured interviews, insightful data were garnered and subsequently subjected to a comprehensive thematic analysis.

Results

The thematic analysis unearthed three core themes: commitment of educators in valuing learner diversity, promotion and understanding of general medicine, and inclusivity and diversity in educational institutions. The first highlighted the centrality of educators’ sincerity, emphasizing the importance of genuine, sustained interactions that foster mutual respect and collaborative learning. The second theme underscored the need to elucidate the intrinsic value and modern-day relevance of general medicine, emphasizing its deep roots in community-based practices and its continuity with long-standing medical traditions. The third theme spotlighted the crucial role of comprehensive medical education in fostering enriching dialogues, embracing varied learning experiences, and capitalizing on the distinctive strengths of educational institutions.

Conclusion

These findings underscore the pivotal shift required in pedagogical approaches to comprehensive medical education. A genuine collaborative educator-learner relationship, the reframing of general medicine’s significance rooted in community welfare, and a strong emphasis on inclusivity and dialogue form the cornerstones of these insights. This study provides a touchstone for restructuring educational strategies, aiming for a more integrated, genuine, and encompassing framework that is particularly vital for the effective propagation of general medicine in regions such as Japan.

## Introduction

Medical education, especially in general medicine, holds significant importance in today’s global healthcare system [1,2]. As the global population ages, there’s an increasing demand for comprehensive care, pushing medical institutions to evolve [3,4]. General medicine, which focuses on managing complex patient conditions from a biopsychosocial perspective, parallels family medicine and general practice [2]. In this context, Japan, one of the most rapidly aging societies, faces a unique challenge [5]. The proficiency of healthcare professionals in general medicine can profoundly shape community care quality nationwide [2], emphasizing the need to continually assess and refine medical education in this domain.

Yet, effective medical education isn’t just about revamping curricula. It should also align with the aspirations, interests, and perceptions of its core recipients: the medical students and residents [6]. Past studies in Japan indicate that existing medical education may not accurately represent the realities of general medicine, primarily due to a shortage of educators and tangible experiences in the field [7]. Students express a desire for more hands-on exposure to general medicine to shape their professional visions [6,8]. Thus, educational institutions should aim to meet these expectations for optimal results.

While the necessity to tailor medical education based on student interests is recognized, specific research detailing this alignment, especially in Japan, remains scarce. What aspects of general medicine education resonate most with students? How do educational frameworks, particularly those concentrating on rural Japanese areas, align with their preferences? Prior research indicates that students are attracted to the comprehensive nature of care and broad scope of general medicine [6,7,9]. Still, the exact expectations of prospective general medicine practitioners regarding their education remain underexplored.

Our study aims to illuminate medical students’ and residents’ aspirations and perceptions concerning general medicine educational institutions. We seek to depict a detailed image of the “ideal” general medicine education in rural Japan, aspiring that such insights will direct educational strategies across the country.

## Materials and methods

Setting

This study took place at Unnan City Hospital, Unnan, Japan. The hospital offers community-based medical education (CBME) to students and residents from various institutions and provides a diverse range of clinical exposure in different care settings [10]. Before joining, students and residents typically undergo a month’s training in rural general medicine at other facilities. However, students observe under medical teachers, while residents can independently assess patients but must consult before prescribing or ordering tests [11,12]. Unnan City Hospital hosts 40-50 trainees annually in their Department of General Medicine [13].

Participants

Between April 2021 and March 2023, 69 trainees (53 medical students and 16 junior residents) undertook the CBME curriculum at Unnan City Hospital. This curriculum aims to instill essential competencies in general medicine as recognized by Japan [14]. Using purposive sampling, those expressing a motivation toward general medicine were informed about the study. In total, 22 medical students, three junior residents, and eight general medicine residents consented to participate.

Data collection

Ethnography

Two researchers, RO and TK, engaged in participatory observation [15]. They observed the daily interactions and learning experiences of the participants. Weekly discussions between RO and TK allowed reflection and alignment on observations. At the end of the curriculum, participants shared their learning outcomes, which were further discussed with RO and TK.

Semi-structured Interviews

On their last day, participants were interviewed by RO using a guide exploring their perspectives on general medicine education. Each session, spanning 30-60 minutes, was recorded, transcribed, and subsequently reviewed with TK.

Analysis

An inductive thematic analysis was employed [16,17]. Initial coding was based on field notes, which were then refined through discussions between RO and TK. Concepts and themes were iteratively developed, with triangulation employed for validity. The analysis was performed throughout the study duration, aiming for theoretical saturation. The final theme was agreed upon by all team members (RO, TK, and CS).

Reflexivity

The research output was co-constructed by participants and researchers. The research team brings varied expertise in rural medical education, ensuring a multi-faceted understanding of the data. To avoid bias, regular team discussions were held to challenge and refine interpretations.

Ethics approval

All procedures adhered to the Declaration of Helsinki guidelines. Participants gave written consent before engagement. The Institutional Ethics Committee of Unnan City Hospital approved the study (approval code 20230014). Confidentiality and anonymity of participants were maintained throughout.

## Results

Results of the thematic analysis

Three major themes emerged from the thematic analysis of field notes and semi-structured interviews: (1) commitment of educators in valuing learner diversity, (2) promotion and understanding of general medicine, and (3) inclusivity and diversity in educational institutions (Table 1). 

**Table 1:** Results of the thematic analysis

Theme	Summary
Commitment of educators in valuing learner diversity	Participants emphasized the value of educators showing genuine interest in their students and their educational journeys. Effective communication and mutual respect between learners and educators were seen as pivotal. The ongoing commitment of educators to engage with learners was also highlighted.
Promotion and understanding of general medicine	The novelty of general medicine as a specialty in Japan led to its lesser-known importance among learners. A need to clearly define and articulate its significance was stressed, along with the necessity to highlight its roots and long-standing practices in community-based healthcare.
Inclusivity and diversity in educational institutions	A significant focus was on the role of institutions in fostering dialogues and interactions among learners. The individual strengths of educational institutions should be recognized and utilized in delivering comprehensive medical education. Concerns were also raised about potential conflicts and the need for collaboration over competition.

**Figure 1:**
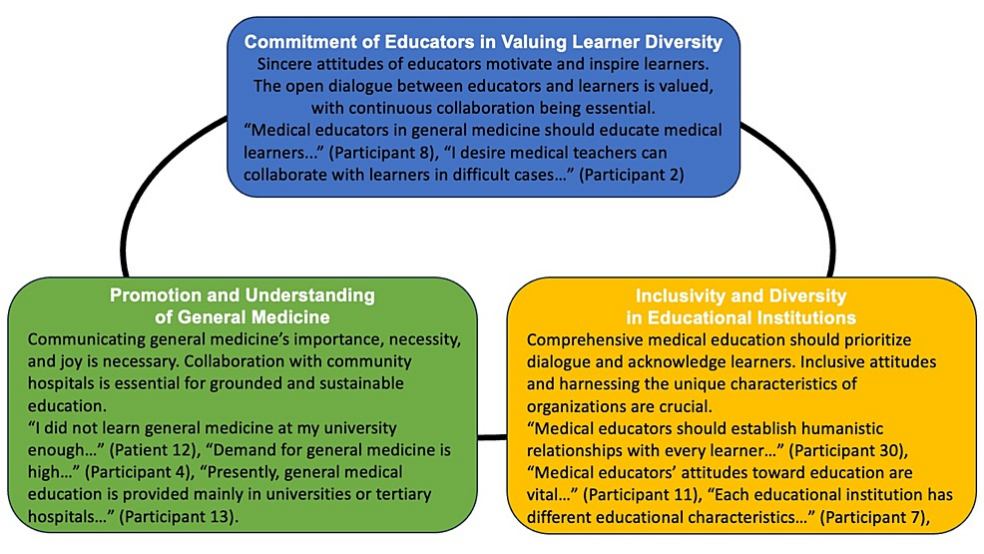
Conceptual figure of the thematic analysis

Figure 1 shows the conceptual figure of this study (Figure 1).

Commitment of educators in valuing learner diversity

At the heart of comprehensive medical education, participants overwhelmingly emphasized the role of educators’ sincere engagement with learners. According to them, a successful educator isn’t just someone who imparts knowledge but genuinely values the diversity and individuality of every learner they engage with. The participants observed distinct variations in motivations and commitments among educators. This led them to believe that only those educators who were genuinely committed and displayed palpable enthusiasm could instill a profound interest in the students. Participant 8 insightfully shared, “Just as a patient can sense a doctor’s genuine concern, we as learners can easily gauge the fervor and dedication of our educators.”

The emphasis was not solely on the educators’ knowledge or skill set but also on their capability to foster open communication and cultivate an environment of mutual respect and collaboration. It was evident that learners yearned for continuous dialogues and engaging discussions, which they found pivotal for their academic growth and inspiration. Participant 2 voiced a shared sentiment, “The intricate cases in medicine often pose challenges. I genuinely hope educators collaborate with learners in these scenarios, as diving deep into these discussions, exploring possibilities, and finding solutions collaboratively can be an invaluable learning experience and a significant motivational booster.”

Promotion and understanding of general medicine

As general medicine emerges as a new specialty, participants discerned a palpable gap in its comprehensive understanding among medical students and residents. They observed that this was not just about grasping medical concepts but about understanding the profound role and potential of general medicine in reshaping healthcare landscapes. Participants were particularly vocal about the necessity for experienced doctors to transmit their vast reservoir of tacit knowledge, experiences, and insights effectively to the next generation.

Moreover, there was a clarion call for educators to elucidate the evolving role, significance, and demand for general medicine, especially within the context of contemporary and future Japanese society. Participant 4 poignantly remarked, “The soaring demand for general medicine is apparent, but the intricate nuances, its holistic importance, and its transformative potential might remain elusive to many learners. This calls for an immersive and comprehensive pedagogical approach.”

Inclusivity and diversity in educational institutions

A recurring theme in the participants’ responses was the profound importance of inclusivity within comprehensive medical education institutions. They fervently believed that these institutions should not just be places of academic pursuit but should serve as crucibles where every learner feels valued, heard, and engaged. Facilitating proactive dialogues between learners and educators was seen as a cardinal step toward this direction.

Furthermore, the participants highlighted the untapped potential in harnessing the distinct strengths, characteristics, and expertise of diverse educational institutions. However, shadows of concerns loomed large, especially regarding the perceived friction or competition among educators. As Participant 28 wisely summed up, “In the grand tapestry of medical education, every thread, every institution has its unique hue and value. Instead of overshadowing each other, we should aim for collaboration and mutual enrichment. Observing such a collaborative spirit and ethos can serve as a beacon, guiding and motivating learners toward excellence.”

## Discussion

This qualitative study offers insights into the ideals surrounding medical education, specifically in Japan’s general medicine realm. The heart of these findings revolves around educators’ earnestness, engagement with learners, and the profound need for mutual respect for individual differences, which are paramount to the learners’ growth trajectory.

An overarching theme was the perceived need for general medical educators to remain cognizant of current societal needs, engage in continuous dialogue with learners, and collaborate on innovative solutions. This sentiment aligns with an expanding body of research that underscores the importance of learner-centered education [18]. The transition from a predominantly didactic approach to a more collaborative, partnership-driven model is evident [19]. Such a model delivers knowledge and nurtures an environment conducive to producing confident and adept practitioners [20].

As general medicine gains prominence, articulating its core principles and indispensable role in healthcare becomes more critical. It plays a crucial bridging role between community and tertiary care settings, demanding a clear exposition of its value proposition to budding medical professionals [21,22]. This study underlines that general medicine isn’t merely a passing trend; it embodies age-old practices centered on continuity, tradition, and a deep-rooted connection with communities [23]. Such insights counteract misconceptions that may undervalue general medicine’s role [24]. Moreover, the preference for more collaborative endeavors between university hospitals and community settings emphasizes the need for an education that resonates with real-world medical scenarios [25].

As highlighted by participants, promoting dialogue-based learning can effectively nurture intrinsic motivation in learners, boosting knowledge retention [26]. Furthermore, leveraging the inherent strengths of diverse educational institutions encourages a spirit of collaboration in what often becomes a competitive academic milieu [27]. Participants’ concerns about possible educator conflicts hint at broader systemic challenges, suggesting the importance of an inclusive, diversified educational strategy [28]. At its core, the principles accentuated in this study are engagement, collaboration, and valuing every learning opportunity, the very essence of general medicine [29].

However, certain limitations must be recognized. Like many qualitative studies, our participant demographics might not encapsulate the broader perspectives of medical educators and learners. The results might echo the biases or specific experiences of our sampled group. Though thematic analysis can provide rich detail, interpretations may introduce inherent biases, and different researchers might discern varying themes from identical data sets. The insights about general medicine in Japan may translate differently to other cultural or geographical milieus, given the diverse norms and views on general medicine globally. The possibility of participants offering socially desirable responses cannot be ruled out. Field notes might not always encapsulate the intricate nuances of participants’ sentiments. Furthermore, as a snapshot of the current state, this study might not capture the dynamic evolution of perspectives in medical education.

## Conclusions

This research highlights the key elements sculpting comprehensive medical education in Japan. It delineates three pivotal themes: educators’ genuine engagement, the necessity to demystify general medicine’s significance, and the role of inclusivity in pedagogy. The study challenges traditional teaching paradigms, highlighting a shift toward more collaborative models. It reinforces general medicine’s long-standing ethos, urging a shift from predominantly university-based training to a more community-centered approach. The call for a diversified, dialogue-driven pedagogy further accentuates the necessity for holistic education. While this study offers invaluable perspectives, they should be interpreted within its intrinsic limitations. Nevertheless, the insights signal a paradigm shift in medical education, propelling it toward a more integrative, genuine, and inclusive framework. This research, with its intricate layers of understanding, sets the stage for future endeavors to refine the quality and reach of medical education, especially in the context of general medicine.
